# Emerging trends of carbon emissions and foreign direct investment: accounting for ecological footprints, renewable energy, globalization, and technological innovations in BRICS

**DOI:** 10.1007/s11356-023-31495-4

**Published:** 2023-12-22

**Authors:** Umar Nawaz Kayani, Ismat Nasim, Ahmet Faruk Aysan, Farrukh Bashir, Umer Iqbal

**Affiliations:** 1 College of Business, Al Ain University, Abu Dhabi, United Arab Emirates; 2 Department of Economics, The Government Sadiq College Women University, Bahawalpur, Pakistan; 3 College of Islamic Studies, Hamad Bin Khalifa University, Qatar Foundation, Ar-Rayyan, Qatar; 4 School of Economics, Bahauddin Zakariya University, Multan, Pakistan; 5 FAST School of Management-National University of Computer and Emerging Sciences, Lahore Campus, Lahore, Pakistan

**Keywords:** Carbon emissions, Foreign direct investment, Ecological footprint, Renewable energy, Globalization, Technological innovations

## Abstract

This paper investigates the intricate interplay between carbon emissions and foreign direct investment within the context of Brazil, Russia, India, China, and South Africa (BRICS) for the period spanning 2000 to 2022. In our comprehensive analysis, we incorporate ecological footprint, renewable energy, globalization, and technological innovations as exogenous variables. Employing a system of simultaneous equations across the BRICS panel, we aim to fully elucidate the proposed relationships. Our empirical findings underscore the following key insights: foreign direct investment, technological innovations, and the adoption of renewable energy sources significantly contribute to the mitigation of carbon emissions in these selected nations. However, it is essential to note that ecological footprints exhibit a positive association with carbon emissions, raising concerns on two fronts: escalating environmental degradation and increased land pressure, both of which contribute to rising ecological footprints in BRICS countries. Additionally, our analysis reveals that foreign direct investment is influenced by its capacity to reduce carbon emissions and bolster renewable energy adoption, while globalization amplifies investment trends within the BRICS nations. To address the environmental repercussions of mining activities, it is imperative to implement stringent control and regulation measures, given their potential adverse impacts, including soil pollution, acid mine drainage, erosion, biodiversity loss, excessive water resource consumption, and wastewater disposal challenges. Nevertheless, proactive steps such as recycling mining waste, adopting environmentally friendly mining equipment, combatting illegal mining, and enhancing overall mining sustainability offer promising avenues to mitigate the environmental footprint of mining operations.

## Introduction

The global economy has experienced remarkable growth and progress over the past two decades, with the participation of developing nations playing a pivotal role in achieving our shared global economic objectives (Xie et al. 2023). Notably, the various economies around the world are now experiencing a heightened flow of financial funds and investments, thanks to increased liberalization (Aysan et al. 2023). However, it is essential to recognize that the effectiveness of foreign direct investment (FDI) and market capitalization varies from one country to another. A substantial body of literature exists on the topic of financial development and its role in optimizing returns from FDI in recipient countries (Choudhry et al. 2023; Kayani et al. 2023a). The seamless movement of capital relies heavily on the efficiency of a nation’s financial system. Thus, our focus should extend beyond merely enhancing net capital inflows; it should also encompass the simultaneous development of robust financial systems. This perspective is supported by both theoretical and empirical research, as demonstrated by Hanif et al. (2019), underscoring the critical role of financial development in fostering economic growth and development. However, it was the innovative research work of McKinnon (1973) and Shaw (1973) that started the debate about the interrelation between growth and institutions of financial development. It is after this research that this topic gained academic attention among research scholars. It was not until the 1990s that the correlation shared between growth and financial development was not very clear since it is mainly dependent on financial liberalization policies. Therefore, it can be harmful to the process of economic development in some cases. Nonetheless, a very positive picture has been shown by research studies that are based on the role economic growth plays in the nexus between FDI and financial development (Wang et al. 2021; Usman and Hammar 2021; Kayani et al. 2023b).

Therefore, the growth of the country which is related to FDI is not only dependent on the investment’s volume. A massive threat was posed to economic development due to 2008’s financial crisis. The aftermath of the crisis is still being faced by many economies all around the world, both developing and developed. It is imperative to understand the financial system’s role so that the risk can be minimized, and capital can be exploited efficiently. Although many different studies are available that address the spillover effect of FDI, the role of FDI in the correlation between growth and economic independence is difficult to find. Therefore, it is considered important to explore the growth-financial development nexus and FDI and capitalization’s role (Wang et al. 2021).

A very crucial role is played by FDI and capital formation in enhancing infrastructure, technology, and industrial progress. However, it has been highlighted by various researchers that financial development of the advanced stage is needed for optimizing the growth that results from FDI. Moreover, the interrelation between financial and economic growth is dependent upon efficiency (Hanif et al. 2019).

It is therefore essential to understand the complex relationship among environmental quality, inward FDI, and economic development which should be the base for developing economically viable policies. It has been observed through literature research that the causal relation existing between environmental quality, inward FDI, and fiscal stability has crucial policy implications which are essentially based on four factors as discussed here (Shao 2018). Firstly, if there is a bidirectional relation between inward FDI, environmental degradation, and economic growth then inward FDI will be a source of promoting economic stability in the host country. This signifies that FDI will help in increasing the funds of a financial system. Consequently, these funds enable the financial markets to develop which also translates into the promotion of economic development. Subsequently, this results in attracting more FDI thereby acting as a vicious cycle. Secondly, economic growth and CO2 emissions’ bidirectional correlation signifies that more CO2 levels will negatively affect economic development and vice versa. Therefore, as countries start developing, the level of environmental degradation also increases; however, environmental degradation tends to be minimized if a specific level of income is attained, under the environmental Kuznets curve (EKC) theory (Abdouli and Hammami 2020).

Thirdly, the existing interrelation between FDI and CO2 emissions signifies that the most vital cause of environmental degradation in the host country is FDI, consequently, as CO2 emissions increase it encourages inflows of inward FDI. Lastly, when considering the interrelation between economic growth and carbon footprint, it is observed that more financing is provided by the financial sector at cheaper rates which are also imperative when considering an investment for projects related to the environment. On the contrary, as financial development increases, so does the emissions of CO2. This signifies that prosperous and well-organized financial mediation seems to be more favorable in assisting the expansion of economic output which also means more consumption of energy and buying bigger emission-intensive products such as automobiles, refrigerators, and washing machines (Cicea and Marinescu 2020).

Exogenous variables such as ecological footprints, renewable energy, globalization, and technical innovations are taken into account in this investigation of the connection between carbon emission (CE) and foreign direct investment (FDI) in the BRICS nations. According to the findings, foreign direct investment (FDI), the use of renewable energy, and ecological footprints all considerably cut carbon emissions (Martins et al. 2023). However, ecological footprints also contribute to environmental deterioration and increasing human strain on land, which affects the energy demand of the BRICS countries. According to the findings of the study, environmental consequences may be reduced by regulating mining operations, recycling trash, encouraging environmentally responsible practices, and tackling illicit mining.

Additionally, FDI can play the role of economic growth’s decisive factor in both the macro and micro-economic arena in some countries (Cicea et al. 2019). Generally, there are two ways for measuring economic progress i.e., gross domestic product (GDP) and the quality of life. In 1990, the major source of financial inflows in the developing economies was FDI. The major attributes of FDI are as follows: it has a lesser degree of volatility and behavior-wise and it is irregular and pro-cyclical. When observed globally, the inflows of FDI have generally increased over time since the 1980s and 1990s and are contributing significantly to the economic development of low-income nations (Simionescu and Naroş 2019). The correlation shared by industrial development, economic growth, and environmental sustainability has been studied by many environmentalists, economists, and corporate researchers. The study is further followed by a review of studies and an overview of EF of BRICS. The “Methodology” section covers the data sources; results are presented in the “Research results” section, and the conclusion, implications, limitations, and policy suggestions are in the “Conclusion” section.

## Literature review

### Globalization and environmental footprint

Increased industrial growth and globalization have instigated a debate regarding its pragmatic and negative impacts on environmental quality and CO2 emissions (Aysan et al. 2020; Kayani and Kayani 2017; Iqbal et al. 2022; Kayani et al. 2023c). Previously published studies available on this topic have classified the impact of globalization on the environment into three major classes i.e., scale, composition, and technique impact. Scale effect refers to the concept that globalization promotes increased financial growth, industrial production, and resource consumption. All these processes require high-energy usage leading to an increase in the global carbon burden. In this way, the scale effect defines the impact of globalization on environmental pollution (Destek and Sarkodie 2019; Tanveer et al. 2022; Usman et al. 2023). According to the consumption impact, the state of industrial development and its dimensions in any country determines the magnitude and degree of impact globalization will likely cast on eco-sustainability (Zaidi et al. 2019; Tanveer et al. 2023). However, typically with the increase in globalization, industrial sectors associated with a limited carbon footprint undergo expansion such as the service industry, while those sectors that possess a high CO2 burden begin to contract. In this way, the net carbon footprint of the country is reduced thus implying no harmful impact of globalization on environmental performance and vice versa. The third impact mechanism is the technique effect that presents different ways by which globalization affects the levels of CO2 emissions in the commercial, residential, and industrial sectors and their influence on ecological degradation. These ways pose diverse impacts on the nature of environmental performance, impact mitigation, and globalization-driven technology modernization or sustainable technology exchange (Lin et al. 2016).

In this regard, the research by Wang and Dong (2019) is significant in this reference which argues that global development positively impacts sustainable technology innovation thus reducing the extent of ecological damage. Various other researchers have explored the influence of globalization and industrial development on pollution and have presented contrasting results. For example, in recent research by Saud et al. (2020), financial development’s role in promoting a sustainable environment in terms of ecological footprint has been researched. The results revealed that globalization reduces adverse environmental impacts considering the case of “One Belt One Road” (OBOR). In addition, the study reported that with the increase in globalization, the development and transfer of sustainable technologies are fostered which not only promotes economic development in a country but also results in a significant reduction in the overall carbon footprint of that country (Hussain et al. 2022; Khalid et al. 2021; Ucan et al. 2023). Consequently, this scenario supports the technique impact of globalization. Similarly, another recent research on the “Generalized Method of Moments” approach scrutinized the correlation shared by energy usage, globalization, and environmental countries in 97 countries (Yang et al. 2020). According to the results, environmental sustainability is positively impacted by the increase in globalization. The findings are by the famous ecological modernization model. Ibrahiem and Hanafy (2020) also focused on investigating the complex nexus between globalization and the selected economic and environmental indicators. These included the per capita income, energy demand, and eco-sustainability in Egypt over more than 40 years from 1971 to 2014. According to the findings, as energy consumption increases, a rise in CO2 emissions is observed whereas globalization results in decreased carbon emissions. Because of the results of the study by Ulucak et al. (2020), a similar correlation exists between globalization and ecological sustainability in nations undergoing economic development.

On the contrary, there is ample literature present highlighting the negative impacts of globalization on environmental protection and providing a relevant evidence base for supporting this narrative. Yilanci and Gorus (2020) recently examined globalization and industrial development’s influence on environmental protection in the Middle Eastern and North African countries. The authors collected data from a set of 14 MENA countries from 1981 to 2016. The results reported that globalization is associated with undermining environmental performance via scale impacts. Similarly, Usman et al. (2020) evaluated the correlation between sustainable power resources, globalization, and ecological footprint and presented that environmental footprint is reduced with the increase in renewable energy utilization. However, environmental pollution has increased with the accelerating globalization rates. This has been attributed to the increased consumption of fossil resources. The study has provided strong evidence in support of the scale effect. In another research, Suki et al. (2020) studied the correlation of globalization and environmental quality in different Malaysian cities for 1970–2018. The results reported a significant positive correlation between globalization and environmental degradation. The reason is the reliance of most industrial sectors on conventional pollution-intensive fossil-driven energy resources like natural gas, petroleum, oil, and coal in Malaysia. Le and Ozturk (2020) also presented similar findings and reported that globalization leads to undermined ecological sustainability. The reason for the positive nexus between globalization and CO2 emissions has been explained by the authors in terms of trade openness and poorly formulated and executed environmental laws.

Ansari et al. (2020) and Saqib et al. (2023) studied the correlation between economic progress, energy demand, and globalization on environmental pollution in terms of the EKC model in Gulf countries including UAE from 1991 to 2017. The findings reported that increased energy usage and a high rate of globalization negatively impact environmental quality in the selected countries. The results implied that the EKC hypothesis fails to hold its validity for GCC countries. In light of all these studies, it can be inferred that there are contrasting opinions concerning globalization’s impact on the quality of the environment, and reaching a consensus is not possible.

### Economic development and environmental pollution

When considering the financial growth and environmental quality nexus, a similar trend is observed where there are two groups of researchers, one in support of the positive nexus while others supporting the negative nexus. According to the group of researchers advocating the influence of increased financial stability in minimizing environmental emissions, economic growth helps in channeling investments in the renewable energy sector. In addition, when a country achieves financial security, it can allocate funds to green businesses, provide subsidies for cleaner production, and facilitate the transfer of sustainable business practices and technologies (Naz and Haider 2023). On the other hand, there is a great deal of literature reporting the role of economic growth in increasing CO2 emissions and associated ecological deterioration. This is defined in terms of the provision of adequate financial and technological resources at an economical cost for expanding previously established industrial enterprises and fostering the development of new ones. More industries mean more environmental pollution and resource consumption. Similarly, high per capita income equips the population to invest in modern electronic equipment or vehicles which in most cases are energy-intensive goods (Cheng et al. 2021). Therefore, economic stability is often associated with a higher carbon footprint as it increases the energy demand consequently leading to environmental deterioration.

The extent and direction of the correlation between financial progressiveness and CO2 emissions also depend significantly upon the political, industrial, and regulatory systems of the country (Charfeddine and Kahia 2019). This can be explained in the light of various studies such as Bayar and Maxim (2020) which employed a regression estimator for observing the economic growth and pollution index correlation. The correlation was observed in 11 countries of the European regions which are currently in the post-transition phase. According to the findings, financial stability is associated with massive energy utilization and increased CO2 pollution confirming a negative correlation between sustainability and economic growth. Similar conclusions have been drawn by Hussain et al. (2020) while examining the contribution of economic prosperity and the high rate of globalization on environmental quality. The environmental quality indicator selected by the study was CO2 emissions. The results reported a positive interrelation between economic growth and carbon footprint. A similar trend was seen between globalization and the selected environmental indicator. This implies that the growing economy is not yielding potential opportunities to shift toward sustainable energy generation and pollution control in the countries under study (Rafiq et al. 2022). Godil et al. (2020) also explored the causality shared by economic stability and eco-footprint in Turkish states over more than 30 years (1986–2018). It has been found that economic development, globalization, and development in the tourism sector negatively impact the environmental quality of the country.

On the contrary, in a recent study, Lahiani (2020) reported an eco-friendly role in economic stability in the country. The study focused on testing the role of financial growth on the quality of the environment in China and reported that the availability of excess economic resources fosters the provision of funds for carrying out projects for environmental protection. Similarly, the investments in green industries also increase which not only augment the productivity of the country but also minimizes the power usage. According to Saidi and Mbarek (2017), GDP growth and a rise in population income lead to higher environmental pollution. However, the introduction of green economic reforms can invert this correlation. Dogan and Seker (2016) investigated the nexus between financial growth and ecological well-being from 1985 to 2011. The findings presented that an increase in economic prosperity leads to comprised environmental quality when viewed in the long term; whereas Zaidi et al. (2019) report that there is an inverse interrelation between economic stability and carbon footprint. This implies the existence of favorable opportunities for development in the eco-sustainability sector by investing in green technologies due to better availability of financial resources. These findings follow the research of Zafar et al. (2019) when considering the case of OECD nations. Thus, it can be inferred that the literature presented a mix of positive and negative views related to financial development’s impact on the environment.

### Overview of the ecological footprints of the BRICS countries

The BRICS cluster includes Brazil, Russia, India, China, and South Africa. The BRICS initiative is a continuation of a legacy that was solidly established in April 1955, when nations from Asia and Africa gathered at the historic Bandung Conference to flex their collective muscles during the Cold War and make their presence felt in the global order. Russia was the organization’s founder. They convened the first BRICS Ministerial Conference on September 20, 2006. During a UN General Assembly session in New York, Vladimir Putin, the president of Russia, requested this gathering. They were all eager to increase their level of international collaboration. The purpose of the study is also to have a look at the ecological footprint of each nation which is an encouraging element of international collaboration. This section of the study is based on the EF with all its sub-sections. As per the Global Footprint Network, the EF is based on the summation of the built-up land, carboncropland, fishing grounds, forest products, and grazing land of each country.

**Fig. 1:**
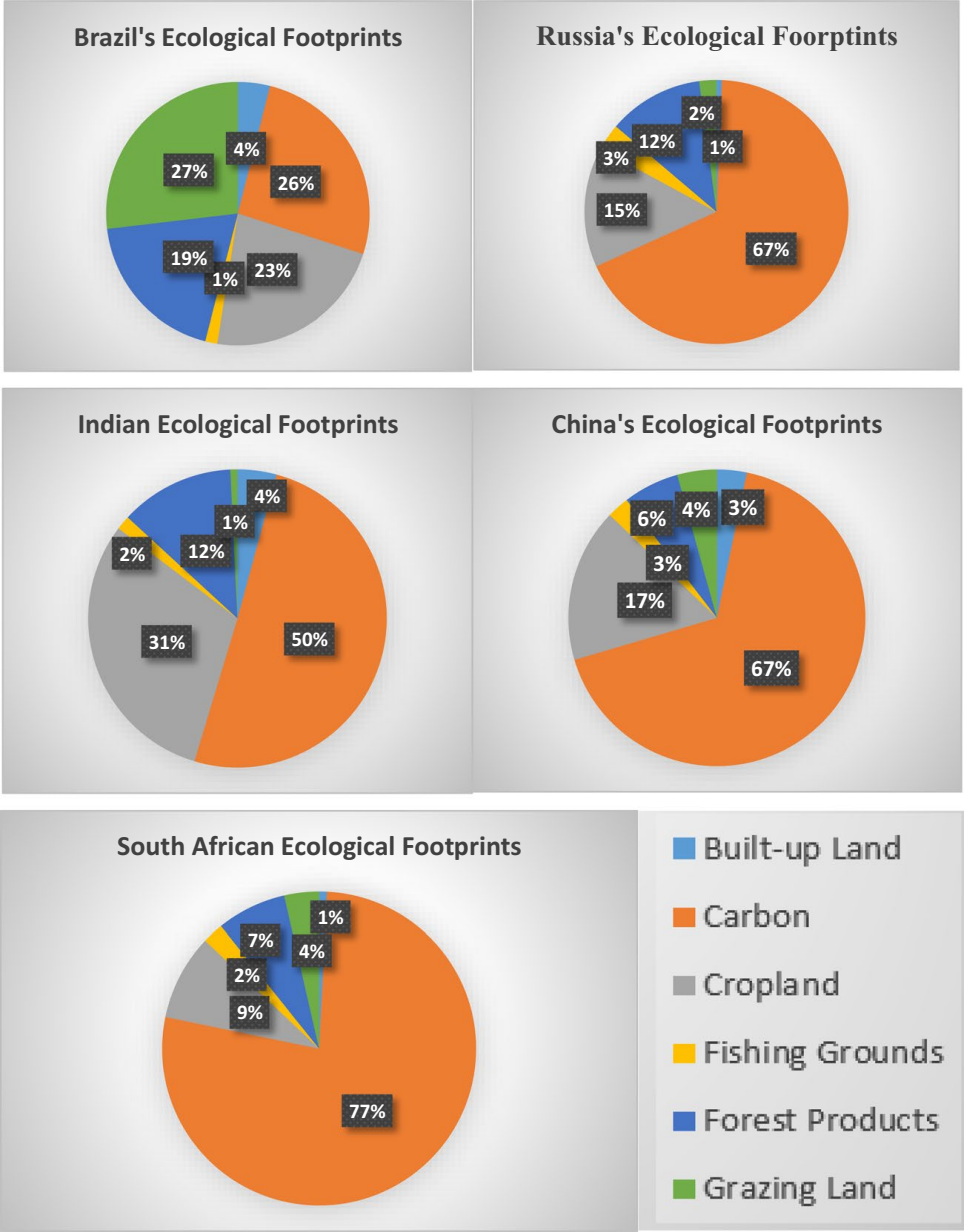
Ecological footprint breakdown. (Date source: Global Footprint Network—the figure is drawn by authors)

Figure 1 and table present the story of EF in all countries of the panel. The numbers tell an interesting story about the breakdown. EF is a measurement of the amount of biologically productive land and water needed by a person, population, or activity while employing current technology and resource management techniques to produce all the resources they need and to absorb the waste they make. Global hectares are often used to calculate the ecological footprint. Trade is international, therefore a person’s or a nation’s footprint includes land and water from all around the globe. The term “ecological footprint” without additional qualification often refers to the ecological impact of consumption. Footprint is a common abbreviation for ecological footprint. Ecological footprint is calculated using a complex set of calculations and models that take into account a wide range of factors. For Brazil, the total EF is 2.721; for Russia, it is 5.199; China has an EF of 3.054; India is among the top ten most polluted countries of the world with 1.040 which is lower than the panel; and South Africa has 3.763. Despite being among the most polluted countries in the world, India has an EF minimum indicating that it has abundant natural resources to bear the pressure of humans on its land. Brazil has rich mining natural resources like tin, iron, and phosphate in addition to large deposits of diamonds, chromium, copper, and many others. Due to heavy mining in Brazil, the EF is relatively higher than in India. Russia has the world’s largest gas resources, oil, and coal reserves. Due to the larger export of these resources, the highest EF is of Russia among the panel. China is the top producer of aluminum, magnesium, talc, cement, coal, gold, graphite, iron, steel, antimony, and many more such products. South Africa also has a variety of minerals.

Cropland, which includes areas used to cultivate food and fiber for human consumption, feed for animals, oil crops, and rubber, is the most bio-productive land-use category. The degree to which farming methods or unsustainable agricultural practices may impact long-term soil deterioration is not yet taken into consideration in current cropland footprint estimations because of a lack of internationally uniform data sets. Crop products utilized for fiber and material production as well as those used as feed for livestock and aquaculture are all included in the farmland footprint. When the values of cropland are viewed, the percentage of cropland is less than a quarter with the second maximum percentage of 23% for Brazil and India with a maximum value of 31%. South Africa has the lowest for the lowest category of cropland that is only 9%.

Grazing land: Livestock is raised on grazing land to provide meat, dairy, hide, and wool. The quantity of livestock feed available in a nation is compared to the amount of feed needed for all animals during that year, with the remaining feed demand assumed to come from grazing land. This comparison yields the grazing land footprint. Table 1 reports the maximum value for Brazil which is 27%, and the remaining countries have values in the range of 1 to 4%.

**Table 1:** Breakdown of ecological footprints for BRICS

	Built-up land*	Carbon*	Cropland*	Fishing grounds*	Forest products*	Grazing land*	EF total
Brazil	0.107	0.709	0.612	0.040	0.521	0.731	2.721
	4%	26%	23%	1%	19%	27%	100%
Russia	0.034	3.512	0.764	0.171	0.607	0.110	5.199
	1%	67%	15%	3%	12%	2%	100%
India	0.044	0.524	0.318	0.015	0.129	0.008	1.040
	4%	50%	31%	2%	12%	1%	100%
China	0.099	2.052	0.512	0.074	0.186	0.131	3.054
	3%	67%	17%	3%	6%	4%	100%
South Africa	0.030	2.914	0.332	0.082	0.270	0.135	3.763
	1%	77%	9%	2%	7%	4%	100%

Table 1 is about the comparative values of EF in BRICS countries. The higher values of various determinants of EF indicate the more pressure of humans on natural resources. India represents the lowest value of EF among the panel and it points out that India is naturally a resource-abundant country, and despite having a large population, its EF is lower. However, Russia is showing the opposite side with the highest value of EF. Source: World ecological footprint by land. Source: Global Footprint Network

Two services are provided by forest land: The quantity of timber, pulp, timber products, and fuel wood used by a nation annually is used to determine the forest product footprint. This component also has a lower rate of percentage with a 19% maximum share for Brazil followed by Russia and India with 12%. The carbon footprint, which reflects the carbon dioxide emissions from using fossil fuels, is also taken into account. Embodied carbon in imported items is also taken into account by the carbon footprint. The area required to trap these carbon emissions serves as a metaphor for it. The quantity of forest area required to absorb these carbon dioxide emissions is used to compute the carbon footprint component of the ecological footprint. The major percentage of humanity’s footprint at the moment is made up of carbon. The forest area and carbon footprint shares are opposite to each other. Brazil with the maximum forest surface has a carbon percentage of 26% which is the lowest in the group. China and South Africa have the lowest surface forests; therefore, they have a higher percentage of carbon which is 67 and 77%, respectively.

Fishing grounds are estimates of the maximum sustainable harvest for several fish species and are used to assess the footprint of fishing grounds. Based on the trophic levels of the individual species, these sustainable catch estimates are transformed into an equal mass of primary output. The world’s continental shelf areas are then separated by this projection of the greatest primary output that may be harvested. Included are fish collected and added to feed mixtures for aquaculture. The percentage of fishing area is the lowest in the overall EF of all countries and it ranges from 1 to 3% only. The built-up land footprint is computed using the amount of land occupied by human infrastructure, including roads, homes, buildings used for industry, and hydroelectric reservoirs. What would have been farmland might now be developed. Like fishing grounds, it has also a small share that ranges from 1 to 4%.

## Methodology

The BRICS countries are our study sample, and we took the data from 2000 to 2022. As the topic of the study has considered the ecological footprints, technological innovations, renewable energy, and globalization as important factors of carbon emissions and foreign direct investment, the data is taken from world development indicators and the global footprint network for BRICS countries. The authors of the study compiled a panel of five countries and obtained the long-run results to depict the relationship of the variables of the study where carbon emissions (CE) and foreign direct investment (FDI) explain each other with some exogenous variables and instrumental variables. The table of the description of variables is as follows:

Table 2 explains the units of measurement and abbreviations of the variables. A foreign enterprise or project receives a foreign direct investment (FDI) from a foreign investor, business, or government (Haider and Tehseen 2022). In other words, foreign direct investment is an investment made by a company with its headquarters in another country that takes the form of controlling ownership in a company, in real estate, or productive assets like factories located in another nation. FDI has the potential to promote and sustain economic development in both the receiving nation and the nation investing. On the one hand, developing nations have supported FDI as a way to fund the development of new infrastructure and the creation of employment for their native workforce. However, global corporations gain from FDI by using it to increase their market penetration abroad. However, one drawback of FDI is that it is subject to many governments’ inspection and regulation, which raises the political risk.

**Table 2:** Description of the variable of the study

Variable names	Abbreviations	Measurements
Foreign direct investment	FDI	Foreign direct investment, net outflows (% of GDP)
Ecological footprints	EF	Ecological footprint (gha)
Globalization	GLOB	KOF Globalisation Index
Carbon emissions	CE	CO2 emissions (kt)
Technological innovation	TI	Research and development expenditure (% of GDP)
Renewable energy resources	RER	Renewable energy consumption (% of total final energy consumption)

This table provides an explanation of the names of the variables that are utilized in the study as well as the data sources for those variables. The variables are also shortened to their acronyms as well, and the research makes use of all of these abbreviations. The study has two indigenous variables that are FDI and CE

To put it simply, ecological footprint (EF) is the result of human activity and is determined by the amount of biologically productive land and water needed to create the commodities we use and ingest the garbage we make. Globalization (GLOB) is the process that makes it easier for people and products to go beyond national boundaries. The integration of markets, commerce, and investments with minimal impediments to hinder the flow of goods and services across countries is primarily an economic notion.

Carbon dioxide emissions (CE), often known as CO2 emissions, are those caused by the burning of fossil fuels and the production of cement. They also include gas flaring and carbon dioxide created during the use of solid, liquid, and gas fuels. An expanded definition of innovation is technological innovation (TI). Although innovation is a fairly well-defined notion, many people—especially those in the academic and corporate worlds—understand it to imply different things. A new or enhanced product or procedure with considerably better technical features is referred to as technological innovation. New items (product innovations) or processes (process innovations) that have been commercialized constitute technologically implemented product innovations.

## Research results

### Description analysis

The descriptive analysis is estimated as a common sample for each variable and is presented in Table 3. The results are for the whole BRICS region. For CE, the descriptive analysis is reported in Table 3. The panel consists of five cross-sections and each cross-section is geographically different in size and population. Table 3 summarizes the carbon dioxide emissions in the panel. The values reported show that EF has a mean value of 3.16 with a standard deviation of 1.42. Similarly, FDI has a mean value of 0.99 of net outflow which is the percentage of GDP with a dispersion value of 1.01. the values of skewness and kurtosis indicated the normality of the panel for all variables. The skewness value is favorable only if it is between 1 and + 1. If the value is higher than 1, it indicates a highly skewed distribution. For the variables of the study, TI has having highly skewed distribution. If the value of skewness is between 1 and ± 0.5, it shows that the distribution is moderately skewed like FDI, GLOB, and CE. However, if the value is between ± 0.5, then it is symmetric like RER and EF.

**Table 3:** Descriptive analysis

	EF	FDI	GLOB	RER	TI	CE
Mean	3.155	0.988	63.655	22.746	1.083	14.056
Median	3.176	0.762	63.888	14.773	1.030	14.263
Maximum	5.788	3.774	72.146	48.920	2.430	16.281
Minimum	0.799	− 2.595	46.308	3.112	0.615	12.559
Std. Dev	1.423	1.015	5.375	16.461	0.433	1.129
Skewness	− 0.030	0.605	− 0.660	0.317	1.475	0.480
Kurtosis	2.053	2.539	1.459	1.477	2.702	2.110
Jarque–Bera	4.311	7.365	9.351	8.037	5.596	8.209
Probability	0.440	0.652	0.154	0.858	0.471	0.378
Observations	115	115	115	115	115	115

Table 3 reports the descriptive statistics of the panel as a whole. All variables of the study are pooled country-wise and then measures of locations (mean, median, and mode) are calculated considering a panel. Skewness and kurtosis are also a part of the descriptive analysis that indicates the distribution of each variable. For robustness, the normality of residuals is estimated for the possibility of misspecification of the model through monitoring the residuals of regression equations. All variables show the acceptance of the null hypothesis which validates the normality of residuals and no misspecification in the model

*EF* ecological footprints, *FDI* foreign direct investment, *GLOB* globalization, *RER* renewable energy, *TI* technological innovation, and *CE* carbon emission

### Correlation matrix

A balanced panel is created from the data for the chosen nations (Luo and Ma 2021). By using a linear trend, the missing data are extrapolated and interpolated. The correlation matrix was acquired for the research to understand the dependency of the variables (Habib and Kayani 2022; Zaman et al. 2018). The results in Table 4 report that there is no issue of multicollinearity. The correlation matrix was created with the aid of the EViews program (Agung 2011), and the results are shown in Table 4.

**Table 4:** Correlation matrix

	CE	EF	FDI	GLOB	RER	TI
CE	1.000					
EF	0.624	1.000				
	0.015					
FDI	0.644	0.535	1.000			
	0.000	0.006				
GLOB	− 0.044	0.682	0.452	1.000		
	0.001	0.002	0.038			
RER	− 0.269	− 0.791	− 0.411	− 0.679	1.000	
	0.008	0.000	0.004	0.000		
TI	0.850	0.245	0.157	0.078	− 0.165	1.000
	0.040	0.031	0.009	0.001	0.000	

Table 4’s results show that there is no correlation between the variables, and the multicollinearity is not severe enough to have an impact on the empirical results. For any variable, the estimated relationships’ directions are expected as per the results of the correlation matrix

*EF* ecological footprints, *FDI* foreign direct investment, *GLOB* globalization, *RER* renewable energy, *TI* technological innovation, and *CE* carbon emission

### Simultaneous equation model

The main variables of the study CE and FDI are explained to each other along with some other

1 $$CE=f\left(FDI, EF, TI, RER\right)$$

2 $$FDI=f(CE, EF, GLOB, RER)$$

Both equations are showing that CE and FDI are dependent variables of the model, and technological innovations, renewable energy, and globalization are exogenous variables, whereas ecological footprints and renewable energy are present in both models as independent variables. According to the above set of equations, the number of equations (G) is two, and we have to check this set of equations for the identification problem first. As per Hall and Asteriou (2016), each equation has one missing variable (M), and as per the identification problem:

$$G-1=M$$

$$G=2\;\&\;M=1$$

So we can say, about the model, that it is exactly identified, and we can proceed further by formulating the equations.

3 $$CE={C}_{1}+{\delta }_{1}FDI+{\delta }_{2}EF+{\delta }_{3}TI+{\delta }_{4}RER$$

4 $$FDI={C}_{2}+{\delta }_{5}EF+{\delta }_{6}CE+{\delta }_{7}GLOB+{\delta }_{8}RER$$

Here, in both equations, *C* represents the constant term, and subscripts 1 and 2 are telling the equations. $\delta$ is representing the coefficients of the exogenous and indigenous variables. For the estimation, EViews statistical software is used for a system of equations. In the system of equations, each equation is written manually according to the simultaneous equation model, and the results for each equation are reported below in Table 5 and 6.

**Table 5:** Results of the first equation

Variables	Coefficients	Std. err	*t*-ratio	Prob
FDI	− 0.272	0.051	5.319	0.000
EF	0.842	0.350	2.407	0.000
TI	− 1.890	0.476	− 3.968	0.000
RER	− 0.065	0.024	− 2.732	0.000
	15.865	3.261	4.865	0.000

Table 5 reflects the results of Eq. 3 where CE is an indigenous variable with a set of exogenous variables. The table indicates the estimated coefficient values in the second column, std. error, *t*-ratios in the next two columns, and probability values in the last column. The probability value is used for indication of the significance of the estimated coefficients, and prob. values declare all variables statistically effective and significant. The estimated signs of the coefficients explain the direction of the relationship of exogenous variables with the dependent one

*EF* ecological footprints, *FDI* foreign direct investment, *GLOB* globalization, *RER* renewable energy, *TI* technological innovation, and *CE* carbon emission

**Table 6:** Results of the second equation

Variables	Coefficients	Std. err	*t*-ratio	Prob
EF	− 0.470	0.113	4.166	0.000
CE	− 0.264	0.083	3.164	0.002
GLOB	0.031	0.024	1.296	0.196
RER	− 0.020	0.009	2.067	0.040
	6.627	2.196	− 3.017	0.003

Table 6 reflects the results of Eq. 4 where FDI is an indigenous variable with a set of exogenous variables. The table indicates the estimated coefficient values in the second column, std. error, *t*-ratios in the next two columns, and probability values in the last column. The probability value is used for indication of the significance of the estimated coefficients, and prob. values declare all variables statistically effective and significant. The estimated signs of the coefficients explain the direction of the relationship of exogenous variables with the dependent one

*EF* ecological footprints, *FDI* foreign direct investment, *GLOB* globalization, *RER* renewable energy, *TI* technological innovation, and *CE* is carbon emission

In Table 5, we showed the results of our first equation of the analysis and set of equations. The results show an inverse and significant prediction of the relationship between CE and FDI. The per-unit increase in FDI will lead to a decrease in CE showing the increasing quality of the environment (Ren et al. 2021; Latief et al. 2021; Apergis et al. 2023; Nasim et al. 2023). The reason behind this positive correlation between FDI and environmental quality is mainly the developed countries intended to allow only such sources of investment that are eco-friendly. The next variable of the system is EF which also presents a direct relation with CE (Ansari 2022; Ansari et al. 2022; Alola et al. 2022). As ecological footprint is accounting the measures of the demand on and supply of nature, it measures the biologically productive area that needs to provide the people’s demand from nature (GF Network 2019; Rees 2018). The addition in EF will decrease the environmental quality when CE increases. The inverse relationship between TI and CE depicts that TI can contribute to the environmental quality of the selected panel with a decrease in CE (Chen et al. 2023; Dong et al. 2022; Irandoust 2016; Ali et al. 2016). The lower rate of association might be due to India as it is the fifth most polluted country in the world. The empirical relationship of RER and CE is also negative which is quite clear. More and more renewable energy will decrease the CE and boost the environment (Saidi, and Mbarek 2016; Sebri and Ben-Salha 2014; Mirza and Kanwal 2017).

The second equation of the system is estimated and reported in Table 6. The negative relation between EF and FDI showed that the lower rate of EF will demotivate and discourage foreign investment. In addition, the FDI with more CE is not welcome in the selected nations to keep the environment clean. The reason behind this notion is simple, natural resources are the main attraction for large investment companies especially when their production is attached to certain natural assets. When the level of such assets becomes smaller, the attraction for such companies may be lower (Chishti 2023; Udemba 2021; Ponce et al. 2023). CE and FDI also move in opposite directions as per the results of the study. Globalization is a boosting factor for FDI as more and more globally integrated economy would be an attraction for the rest of the world. However, the estimated score here is not statistically significant at a very low rate of acceptance. To increase the FDI in the context of globalization, it is a requirement to alter foreign policies and make the FDI more friendly (Aluko et al. 2021; Akadiri and Ajmi 2020; Tiba and Belaid 2020). RER is a discouraging factor for FDI due to the nature of the foreign investment. If a country leads to an increase in the use of renewable energy, then it will act like a restriction on the investors that might be based on traditional sources of energy (Ben Jebli et al. 2019; Ekwueme et al. 2021). The constant term of the equation represents the value of autonomous FDI that is not affected by any of the factors of the study. The value of a constant tells that 6.67 units of FDI are independent of any factor, and this value is statistically significant at a 0.3% level.

## Conclusion

The study intended to investigate the relationship between CE and FDI in such a way that both are explaining factors for each other keeping in view the EF as one of the main exogenous variables along with TI, GLOB, and RER. To obtain the empirical results of the study, simultaneous equations were used with two equations and indigenous variables. The set of equations tested for possible identification and our model becomes an exactly identified model. By using the statistical software EViews, the equations estimated and reported results showed that EF is having a major and positive impact on CE and FDI that is statistically significant as well. The equation for CE is having FDI as a negative determinant along with RER, indicating the need for green finance (Khan et al. 2022). The FDI may deteriorate the environment if the investment is not based on eco-friendly means of production. Similarly, RER is also pushing the CE downward and improving the environmental quality. Technological innovation is still increasing the production of CE due to the presence of India in the panel. India is the 5th most polluted country in the world.

The second equation of the study is projecting the impact of CE, GLOB, and RER on FDI along with EF. The results are not surprising for this equation, as CE and RER are empirically validated as discouraging factors for FDI and GLOB is behaving inversely. A higher rate of EF also pushes the FDI down and depicts the importance of natural resources for any economy.

The feasibility of achieving carbon emissions in the economic and environmental performance of the BRICS nations was investigated, as the study’s title implies. Indicators were carefully chosen depending on their applicability to the study’s goal. Given the complexity of the study and the ecological footprint’s comprehensiveness, which includes six environmental components, it is decided to use it as an indicator of environmental performance (quality of the environment). Other than ecological footprint, the globalization index, technical advancement, foreign direct investment, and renewable energy were thought significant to the goal of this study. The policy emphasis should instead be on improving environmental quality and FDI after taking a serious look at the existing status of the BRICS environment and the huge EF effect. This should be accomplished through raising public awareness of the need to reduce carbon emissions. Several projects have been launched due to technology advancements, and the globe is currently focused on new green construction laws that should be promoted to lower CE. The diversification of energy sources to include more renewable sources is the goal of these programs. Also, a policy should be put into place to mandate the use of energy-efficient items, which are mostly utilized in the housing sector. By providing provisions for expanding accessibility of public mass transit systems like metro and light rail, the transportation sector should be strengthened. The rate of automobile use by the general public, who will favor the mass transit system, will be impacted by this. The rate of fuel and petrol consumption among the families would be affected equally by this.

The BRICS countries must take another step, which they must create and execute in a realistic amount of time with seriousness toward the FDI pattern, which shows that the chosen countries will get more polluted as CE increases. Nonetheless, it is clear that FDI is critical to environmental performance and that nations with more environmental deterioration may choose to use it. This is made feasible by FDI, which introduces favorable technology from wealthy nations. So, to preserve this encouraging trend toward sustainable growth, certain restrictions that are preventative against FDI should be relaxed.

### Implications, limitations, and future directions

The BRICS nations should also concentrate on the supply chain of energy consumption to make it effective and clean, which will convert their impact to negation on the quality of the environment and lower the ecological footprint and carbon emissions, as suggested by Dincer (2000), and sustainability criteria for renewable energy (Popp et al. 2014). Since BRICS will be the largest provider of global renewable capacity, the BRICS nations also need to reconfigure their energy policies following their present geographic, economic, sociological, and environmental values to address environmental challenges (Pathak and Shah 2019). Moreover, strict environmental regulations assist BRICS nations in reducing their ecological footprint; as a result, these nations should maintain strict environmental regulations. The BRICS nations may also increase environmental institutional quality, as indicated by Hassan et al. (2020), who claimed that high institutional quality results in the more effective implementation of environmental rules. There should be control and regulation on the mining of minerals as its higher rates can result in erosion, loss of biodiversity, significant use of water resources, wastewater disposal issues, acid mine drainage, and contamination of soil, and groundwater can be affected by chemicals emitted from mining process (Martins et al. 2022). However, the environmental impacts of mining can be reduced by reusing mining waste, using eco-friendly equipment, shutting down illegal mining, and improving the sustainability of mining.

Also, the BRICS nations should implement a green development strategy and advance technology to reduce economic and environmental quality, as China’s industrial sector’s environmental performance grew by 58% between 1998 and 2009, mostly as a result of technical advancement (Meng et al. 2013). Also, the BRICS nations should be concerned with the industry sector’s efficiency and treatment procedures since some evidence suggests that China’s industrial systems are generally ineffective, particularly in the manufacturing process and pollution removal procedures (Li et al. 2018; Xie et al. 2023). After addressing these concerns, it will assist BRICS nations in achieving economic development that increases industrial sector and energy sector efficiency to fulfill the SDGs and Paris Agreement objectives and to boost BRICS nations’ soft power in global governance (Petrone 2019). It is recommended that further study be done to expand the independent variables, which include the ecological footprint, globalization, technological innovation, and renewable energy characteristics. This study technique may be used for various environmental study topics such as the connections between health and the environment, tourism and the environment, and innovation and the environment, among others.

Attempts to research the BRICS nations could run across obstacles including censorship, prohibitions, or political prejudice is challenging which may be one of the limitations. Another issue is that the analysis of economic trends can be made more challenging by the interconnection of various countries within the global economy. Due to the large differences in data quality and availability among the BRICS nations, it can be difficult to make cross-country comparisons. Furthermore, BRICS states are prone to quick changes on the economic, political, and social fronts; hence, longitudinal studies are required to correctly capture these changes. As a result, those doing study need to manage these issues carefully while also taking into consideration the effect that economic crises have on others.

## Data Availability

Available upon request.
